# Can CT Radiomics Detect Acquired T790M Mutation and Predict Prognosis in Advanced Lung Adenocarcinoma With Progression After First- or Second-Generation EGFR TKIs?

**DOI:** 10.3389/fonc.2022.904983

**Published:** 2022-07-06

**Authors:** Xiaohuang Yang, Chao Fang, Congrui Li, Min Gong, Xiaochun Yi, Huashan Lin, Kunyan Li, Xiaoping Yu

**Affiliations:** ^1^ Department of Radiology, Hunan Cancer Hospital, The Affiliated Cancer Hospital of Xiangya School of Medicine, Central South University, Changsha, China; ^2^ Department of Clinical Pharmaceutical Research, Hunan Cancer Hospital, The Affiliated Cancer Hospital of Xiangya School of Medicine, Central South University, Changsha, China; ^3^ Department of Pharmaceutical Diagnosis, General Electric (GE) Healthcare, Changsha, China

**Keywords:** lung adenocarcinoma, radiomics, acquired T790M mutation, EGFR TKI, prognosis prediction

## Abstract

**Objective:**

To explore the potential of CT radiomics in detecting acquired T790M mutation and predicting prognosis in patients with advanced lung adenocarcinoma with progression after first- or second-generation epidermal growth factor receptor (EGFR) tyrosine kinase inhibitor (TKI) therapy.

**Materials and Methods:**

Contrast-enhanced thoracic CT was collected from 250 lung adenocarcinoma patients (with acquired T790M mutation, n = 146, without mutation, n = 104) after progression on first- or second-generation TKIs. Radiomic features were extracted from each volume of interest. The maximum relevance minimum redundancy and the least absolute shrinkage and selection operator (LASSO) regression method were used to select the optimized features in detecting acquired T790M mutation. Univariate Cox regression and LASSO Cox regression were used to establish the radiomics model to predict the progression-free survival of osimertinib treatment. Finally, nomograms (which) combined clinical factors with radscore to predict the acquired T790M mutation and prognosis were built separately. In addition, the two nomograms were validated by the concordance index (C-index), decision curve analysis (DCA), and calibration curve analysis where appropriate.

**Results:**

Clinical factors including the progression-free survival of first-line EGFR TKIs, EGFR mutation, and N stage and 12 radiomic features were useful in predicting the acquired T790M mutation. The area under the receiver operating characteristic curves (AUC) of clinical, radiomics, and nomogram models were 0.70, 0.74, and 0.78 in the training set and 0.71, 0.71, and 0.76 in the validation set, respectively. The DCA and calibration curve analysis demonstrated a good performance of the nomogram model. Clinical factors including age and first-generation EGFR TKIs and 12 radiomic features were useful in patients’ outcome prediction. The C-index of the combined nomogram was 0.686 in the training set and 0.630 in the validation set, respectively. Calibration curves demonstrated a relatively poor performance of the nomogram model.

**Conclusion:**

Nomogram combined clinical factors with radiomic features might be helpful to detect whether patients developed acquired T790M mutation or not after progression on first- or second-generation EGFR TKIs. Nomogram prognostic model combined clinical factors with radiomic features might have a limited value in predicting the survival of patients harboring acquired T790M mutation treated with osimertinib.

## Introduction

Lung cancer is the second most common malignant tumor and the leading cause of death for both men and women, of which 51% were initially diagnosed with metastasis, leading to a low 5-year survival rate of 21% (1). Non-small cell lung cancer (NSCLC) patients took a major proportion of 76% of all lung cancer patients whose survival has been improved substantially due to the development of target therapy (2).

It was reported that more than 50% of the Asian population with NSCLC harbor epidermal growth factor receptor (EGFR) mutation (3). For advanced EGFR-mutated NSCLC, epidermal growth factor receptor tyrosine kinase inhibitors (EGFR TKIs) represent the standard of care as first-line treatment and help to reduce mortality and improve survival (2). EGFR TKIs include first generation (for example, gefitinib, erlotinib, and icotinib), second generation (for example, afatinib and dacomitinib), and third generation (for example, osimertinib, aumolertinib, avitinib, and rociletinib). First- and second-generation TKIs target sensitizing EGFR mutation, while third-generation TKIs target both sensitizing EGFR mutation and resistant exon 20 T790M mutation. As reported, osimertinib is not cost-effective for first-line treatment (4). Still, a substantial number of EGFR-mutated NSCLC patients choose first- or second-generation EGFR TKIs as first-line treatment. Unfortunately, these patients will develop resistance to TKIs inevitably with a duration of 9–12 months (5). The mechanism of resistance lies in acquired T790M mutation, MET amplification, and histological transformation to small cell lung cancer, taking a proportion of 50%–60%, 20%, and 5%–10%, respectively (6). Patients with acquired T790M mutation can benefit from third-generation EGFR TKIs; however, patients without acquired T790M mutation have to seek other treatment options such as platinum-based chemotherapy. To further confirm the mechanism of resistance, biopsy to obtain a tissue sample is a necessity. This invasive procedure may have an underlying risk of getting an unsatisfied tissue sample and requiring more attempts. Although liquid biopsy is a much easier and less risky procedure to conduct, the blood test sensitivity was 75% (7). A non-invasive procedure to select patients who harbor acquired T790M mutation and will thus benefit from osimertinib treatment after progression on first-line TKIs is of great clinical importance.

Radiomics turns medical images into quantitative data and links it to the diagnosis, treatment, and prognosis of lung cancer. Several studies showed promising results of radiomics in the detection of EGFR mutation and survival prediction in patients with advanced lung cancer treated with EGFR TKIs (8–10). To the best of our knowledge, very few reports aimed at using radiomics to detect acquired T790M mutation, and only one study explored the prognosis prediction of CT radiomics in metastatic NSCLC with T790M mutation receiving osimertinib treatment (11, 12). Therefore, this study aimed to explore the utility of CT radiomics in selecting candidates who will benefit from osimertinib treatment after progression on first- or second-generation EGFR TKIs, as well as in identifying patients who were likely to have a longer duration of osimertinib treatment.

## Materials and Methods

### Patients

**Figure 1:**
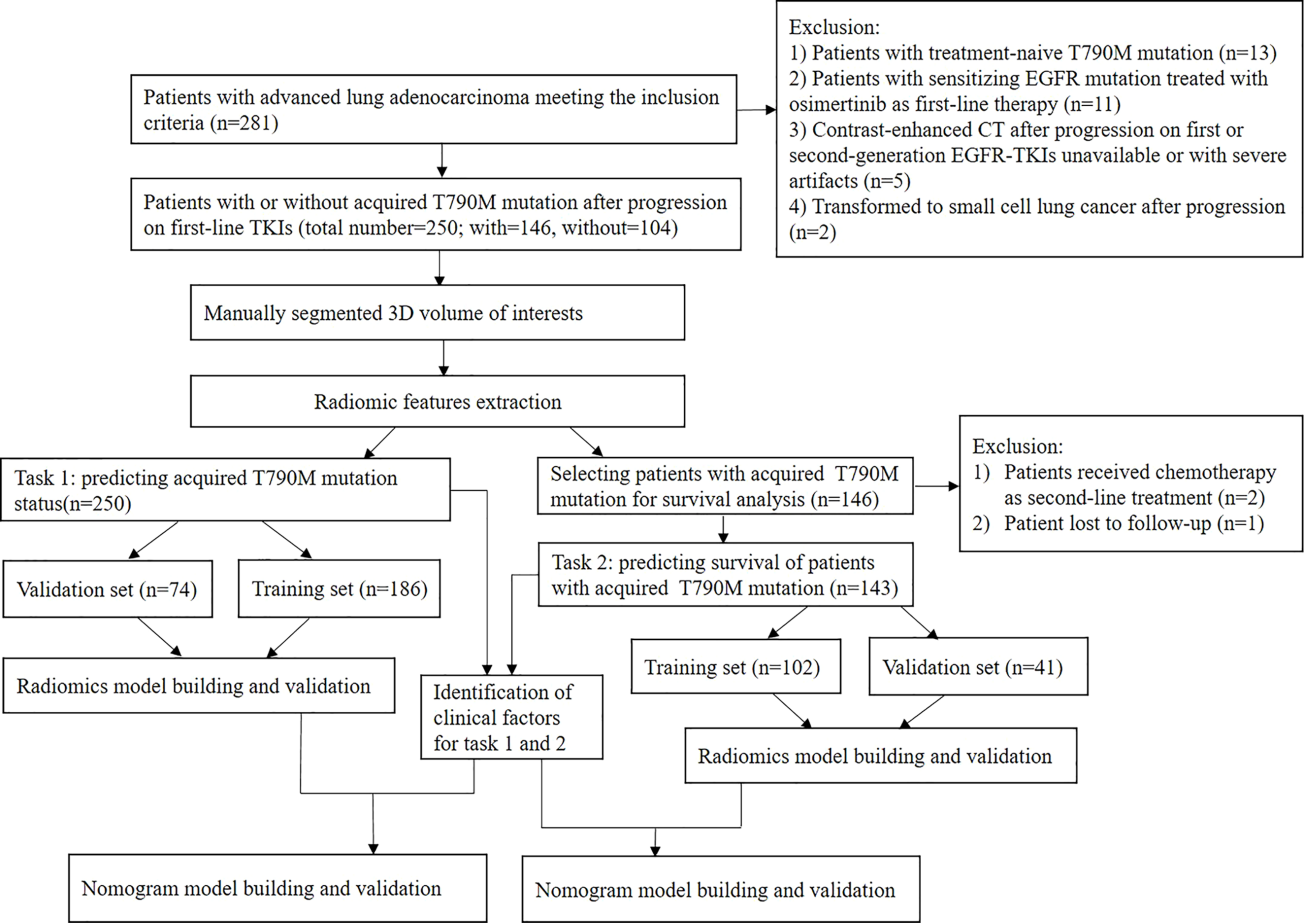
Flowchart of the study.

This study was approved by the medial ethnical committee of Hunan Cancer Hospital, with a waiver of patients’ written consent. The patients hospitalized in our hospital between October 2013 and October 2020 was retrospectively analyzed. The patients’ clinical, pathological, and molecular test data were collected from an electronic medical record system. Inclusion criteria included: 1) histologically or cytologically proved advanced lung adenocarcinoma harboring sensitizing EGFR mutation treated with first- or second-generation EGFR TKIs as first-line therapy; 2) imaging-proven progression on first- or second-generation TKIs; 3) patients underwent chest contrast-enhanced CT at the time of confirmed progression, and the interval between CT and confirmed progression was within 3 days; 4) patients underwent a tissue or liquid biopsy procedure to test the EGFR mutational status after progression, and the interval between biopsy and confirmed progression was within 2 weeks; 5) clear molecular results of T790M mutation; and 6) the clinical, radiological, pathological and molecular test data were intact. Exclusion criteria included: 1) patients with treatment-naive T790M mutation; 2) patients with sensitizing EGFR mutation but chose the third generation of EGFR TKIs as first-line treatment; 3) CT images were not accessible or with severe artifacts; or 4) histological transformation to small cell lung cancer. It is worth noting that in order to avoid false-negative T790M results caused by liquid biopsy, patients without T790M mutation were confirmed only by a tissue sample (histology or cytology), and patients with T790M mutation were confirmed either by a tissue sample or blood sample. **Figure 1** shows the flowchart of the study.

### Follow-Up and Progression-Free Survival

The follow-up interval was 9 weeks for patients treated with first- or second-generation EGFR TKIs. For patients treated with osimertinib after first-line EGFR TKI failure, the therapeutic response was evaluated 4 weeks after the initiation of osimertinib treatment, and the subsequent follow-up interval was 8 weeks. Chest and upper abdominal CT was routinely performed, head MRI and neck and pelvic CT were selectively performed according to each individual’s condition. The TNM stage was assessed by the 8^th^ edition American Joint Committee on Staging System (AJCC) (13). Progression-free survival (PFS) for first-line EGFR TKI therapy was defined as the time from the initiation of EGFR TKI–targeted therapy to the date of the confirmed disease progression. PFS for second-line therapy was defined as the time from the initiation of osimertinib therapy or chemotherapy to the date of the confirmed disease progression, and PFS was censored at the date of the last follow-up, or the date of quitting because of severe side-effects. The duration of PFS was assessed by investigators according to the Response Evaluation Criteria in Solid Tumors, version 1.1 (14).

### Biopsy Procedures and Mutation Analysis

Histology samples (including tissue biopsy and cytology samples) or peripheral blood samples were used for EGFR T790M mutation analysis. Tissue samples were obtained from the primary lung tumor or accessible metastases with the guidance of CT or Ultrasound (US) or with the assistance of endobronchial US (EBUS) or bronchoscopy. Cytology samples were obtained from thorax effusion. The methods of EGFR T790M mutation analysis were droplet digital PCR (ddPCR) or next-generation sequencing (NGS).

### CT Image Acquisition and Segmentation

The chest images were obtained from four CT scanners (Philips Brilliance 16-detector, Siemens Somatom Definition AS^+^ 64-detector, United Imaging uCT760 64-detector, and GE Revolution 128-detector), with a breath-held helical acquisition, 120- or 140-kV tube voltage, 160–240 mAs tube current, 0.625–1.0 pitch, 0.625–1.0-mm collimation, 5-mm section thickness, 350–435-mm field of view (FOV), and 512 × 512 matrix. Iodinated contrast media (Iohexol) was administrated intravenously at a rate of 3 ml/s and a dosage of 1.5ml/kg body weight, with a 60-s delay.

All chest images were reconstructed by using a standard lung reconstruction algorithm, with a slice thickness and interval of 1.0 mm. For radiomics analysis, only thin-thickness contrast-enhanced images from the lung reconstruction algorithm were used.

The axial CT images in digital imaging and communication in medicine (DICOM) format were exported from the picture archiving and communication system. Two radiologists (radiologists A and B, with 5 and 8 years of experience in chest radiology, respectively),who were both blind to the clinical, pathological, and molecular test results but were optional to know the location of primary lung lesions, performed lesion segmentation independently using the ITK-SNAP (Version 3.6, www.itksnap.org) software. Lesions were delineated on the inner edge layer by layer. To better define the tumor border, contrast-enhanced images in the mediastinal window were read when performing tumor segmentation. Firstly, 50 randomly chosen lesions were separately segmented by the above-mentioned radiologists A and B at the same time. A week later, radiologist B repeated the segmentation. Intra- and interobserver correlation coefficients (ICCs) were calculated. As good ICC results (ICC>0.75) were obtained in the present study, all the rest images were segmented by radiologist B. These eventual ROIs were automatically converted to the volume of interests (VOIs) and saved as an nifti (NII) format file.

### Feature Extraction and Radiomics Construction

Before extracting the radiomic features, spacing 1*1*1 was performed to ensure that all the CT images had the same resolution. Meanwhile, gray-level standardization was applied to reduce the gray-level differences caused by the imaging procedure. Then, normalization with the final 256 bins was performed using the gray-scale discretization method to remove the potential differences of CT images acquired from different CT scanners.

A total of 396 radiomic features were extracted from each VOI *via* AK software (Life Sciences, General Electric Healthcare, USA). These features were involved with six categories, including the histogram, texture parameters, form factor, gray-level co-occurrence matrix, gray-level run-length matrix, and gray-level zone size matrix.

To select radiomic features useful in detecting an acquired T790M mutation, two-step methods, including the maximum relevance minimum redundancy (mRMR) and the least absolute shrinkage and selection operator (LASSO) regression method were used. Firstly, mRMR was performed to eliminate the redundant and irrelevant features. Then LASSO method using 10-fold cross-validation was conducted to choose the optimized subset of features to construct the final model. To select radiomic features useful in predicting survival, univariate Cox regression and LASSO Cox regression were used. The radiomics signature (radscore) was created by a linear combination of selected features with non-zero coefficients.

The patients were randomly divided into training and validation sets in a proportion of 7:3. Univariate and multivariate logistic analyses were carried out to screen the clinical variables that showed a relation to predict acquired T790M mutation. Univariate and multivariate Cox regression analyses were used to select clinical variables that help to predict prognosis. Variables with a *P*-value greater than 0.05 in univariate analysis were obsoleted. Then, the significant clinical variables and radscore were introduced into the stepwise multivariate logistic regression analysis to build the combined nomogram model.

### Statistical Analysis

Statistical analysis was conducted by R software (Version 3.6.0, http://www.Rproject.org). A two-tailed *P* < 0.05 was considered statistically significant. Independent t-test, chi-square test, or Fisher exact test was performed to analyze the differences in continuous or category variables, respectively. Univariate and multivariate Cox logistic regression was applied to select clinical variables that help to predict prognosis. The Pearson correlation coefficient was used to evaluate the intra- and inter-observer reproducibility. The receiver operator characteristic (ROC) curve was applied to evaluate the classification efficacy. The comparison of ROC curves among different models was performed by DeLong’s test. The discrimination of each survival prediction model was quantified by the C-index and 95% confidence interval (CI). The clinical significance of the individual prediction model was evaluated by the decision curve analysis.

## Results

### Patients’ Characteristics

A total of 250 patients with advanced lung adenocarcinoma (stage IIIc, n = 2; stage IVa, n = 92; stage IVb, n = 156) after progression on first- or second-generation EGFR TKIs were enrolled in our study. There were 146 patients with acquired T790M mutation and 104 patients without acquired T790M mutation. The baseline characteristics at biopsy after first- or second-generation EGFR TKI failure are summarized in **Table 1**.

**Table 1:** Baseline characteristics at biopsy after progression on first-line epidermal growth factor receptor tyrosine kinase inhibitors.

	Training set (n=176)	Validation set (n=74)	*P*-value
Age (y)	56.8 ± 9.2	55.9 ± 10.0	0.465
Sex			
Male	78	34	
Female	98	40	0.923
Smoking history			
With	56	25	
Without	120	49	0.877
ECOG Performance status			
0	25	13	
1	146	60	
2	5	1	0.640
EGFR mutation			
Exon 19 deletion	93	41	
Exon 21 L858R	73	31	
Others	10	2	0.600
EGFR TKI			
Icotinib	85	37	
Gefitinib	46	24	
Erlotinib	38	10	
Dacotinib	2	2	
Afatinib	5	1	0.431
Treatment response			
CR	3	1	
PR	119	44	
SD	54	29	0.426
PFS (m)	16.1 ± 9.5	15.9 ± 9.8	0.874
T stage			
T1–2	83	38	
T3–4	93	36	0.641
N stage			
N0-1	60	28	
N2–3	116	46	0.674
M stage			
M0	1	1	
M1a	43	18	
M1b	24	6	
M1c	108	49	0.589
TNM stage			
IIIc	1	1	
IVa	68	24	
IVb	107	49	0.552
Tumor differentiation			
Well differentiated	2	2	
Moderately differentiated	46	19	
Poorly differentiated	76	32	
Unknown	52	21	0.843
Radscore	0.4 [0.1, 0.7]	0.4 [0.0, 0.9]	0.761

CR, complete response; PR, partial response; SD, stable disease; PFS, progression- free survival; ECOG Performance status, Eastern Cooperative Oncology Group Performance status.

Among the 250 patients, 210 remained for survival analysis, including 143 patients with acquired T790M mutation who had received 80 mg osimertinib once daily and 67 patients without T790M mutation treated with a 3-week cycle of platinum-based chemotherapy. Of 146 patients with T790M mutation, 3 were ruled out because of being lost to follow-up (n=1), or receiving chemotherapy as second-line treatment (n = 2). Of 104 patients without T790M mutation, 37 were ruled out because of being lost to follow-up (n = 31) [The detailed reasons of patients lost to follow-up were listed as follows: follow-up time less than a month (n = 14); treated with traditional Chinese medicine, immune checkpoint inhibitors, or angiogenesis inhibitors but without a regular imaging checkup (n = 12), and transferred to other hospitals (n = 5)] or sticking to first-generation TKIs after progression (n = 6).

### Different Models in Differentiating Patients With or Without Acquired T790M Mutation

#### Clinical Model

The univariate logistic analysis showed that the PFS of first-line TKIs (OR: 1.06, 95%CI: 1.02–1.11, *P* = 0.003), EGFR mutation (OR: 0.51, 95%CI: 0.30–0.85, *P* = 0.009), and N stage (OR: 0.48, 95%CI: 0.25–0.92, *P* = 0.028) served as the risk factors of acquired T790M mutation. Multivariate logistic analysis revealed that the above-mentioned three clinical factors remained to be independent predictors of acquired T790M mutation. Those with acquired T790M mutation tended to have longer PFS (18m for the group with mutation vs. 13.4m for the group without, *P* = 0.001), lower N stage (42 for the group with mutation vs. 18 for the group without, *P* = 0.039), and were more likely to harbor initial 19 exon deletion (61 for the group with mutation vs. 32 for the group without, *P* = 0.015) in the training set. However, between the group of patients with T790M mutation and the group without mutation, no statistical significance was found in the PFS of TKIs (17.2m for the group with mutation vs. 13.9m for the group without, *P* = 0.149), N stage (19 patients with lower N stage for the group with mutation vs. 9 patients with lower N stage for the group without, *P* = 0.279) and the initial EGFR mutation (27 patients with exon 19 deletion for the group with mutation vs. 14 patients with exon 19 deletion for the group without, *P* = 0.115) in the validation set.

#### Radiomics Model

Among 396 features, mRMR was firstly used for dimension reduction, and 30 features were retained. Then, 12 optimized features were selected by LASSO regression and were ultimately introduced into the radiomics model. LASSO regression was displayed at **Supplementary Material 1**. The formula for calculating radscore is displayed as follows:


$$\begin{array}{c} \text{Radscore }=\;-0.205*\text{GLCMEntropy}\_\text{angle}45\_\text{offset}7+\\ 0.286*\text{LongRunHighGreyLevelEmphasis}\_\text{angle}135\_\text{offset}7+\\ 0.192*\text{Compactness}2+\\ 0.031*\text{LongRunLowGreyLevelEmphasis}\_\text{AllDirection}\_\text{offset}7\_\text{SD}+\\ 0.114*\text{ShortRunEmphasis}\_\text{AllDirection}\_\text{offset}4\_\text{SD}+\\ 0.033*\text{Correlation}\_\text{AllDirection}\_\text{offset}7\_\text{SD}+-\\ 0.359*\text{RMS}+-\\ 0.194*\text{GLCMEntropy}\_\text{AllDirection}\_\text{offset}1\_\text{SD}+-\\ 0.362*\text{LowIntensitySmallAreaEmphasis}+\\ 0.362*\text{Correlation}\_\text{angle}90\_\text{offset}7+\\ 0.191*\text{ShortRunEmphasis}\_\text{angle}45\_\text{offset}1+\\ 0.273*\text{ClusterProminence}\_\text{angle}45\_\text{offset}7\;+\;0.358 \end{array}$$

The median of radscore was significantly higher for the group of patients with acquired T790M mutation than the group without mutation, both in the training set (0.6 vs. 0.2, *P* < 0.001) and the validation set (0.6 vs. 0.1, *P* = 0.002). DeLong’s test showed that the AUC between radiomics and clinical models exhibited no statistical significance in the training set (*P* = 0.375) or in the validation set (*P* = 0.980), indicating that radiomics alone can achieve a similar performance as the clinical model does.

#### Nomogram Model

The formula for calculating the nomoscore was presented as follows:


$$\begin{array}{c} \text{Nomoscore }=\;\left(\text{Intercept}\right)*1.141+\text{ PFS}\_\text{of}\_\text{first}\_\text{line}\_\text{EGFR}\_\text{TKIs }*0.044+\\ \text{EGFR}\_\text{mutation}*-0.578+\text{N}\_\text{stage}*-0.679+\text{Radscore}*1.558 \end{array}$$

**Figure 2:**
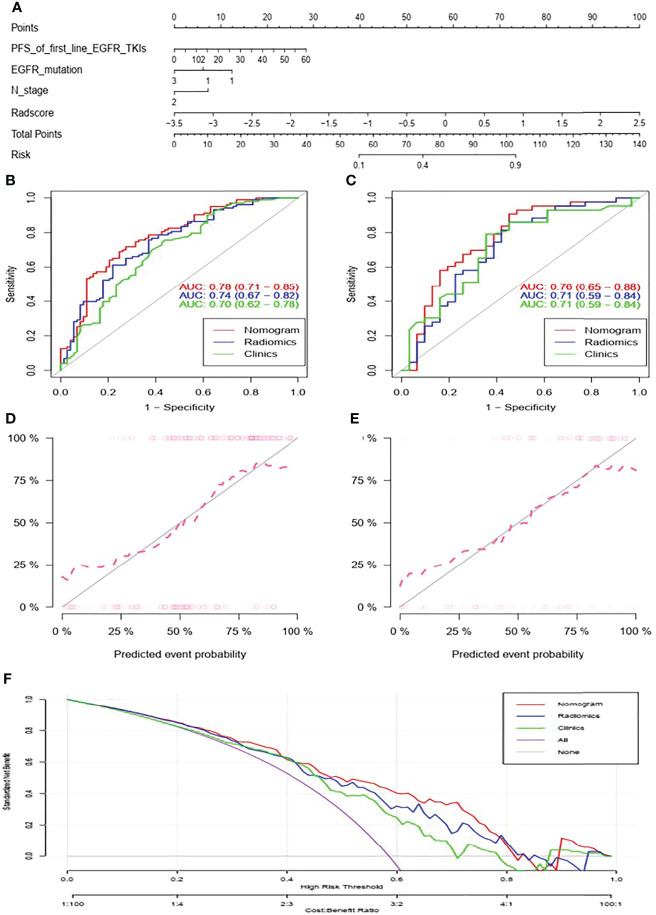
Nomogram for predicting acquired T790M mutation in advanced lung adenocarcinoma patients after progression on first- or second- generation EGFR TKIs **(A)**. ROC curves of the clinical, radiomics, and nomogram models in the training set **(B)** and in the validation set **(C)**. Calibration curve of the nomogram in the training set **(D)** and in the validation set **(E)**. Decision curve analysis of clinical, radiomics, and nomogram models **(F)**.

The nomogram for predicting acquired T790M mutation is displayed at **Figure 2A**. As for predicting patients with or without acquired T790M mutation, the AUCs of the clinical, radiomics and nomogram models were 0.70, 0.74, and 0.78 in the training set, respectively **(** **Figure 2B** **)**. In the validation set, the corresponding AUCs were 0.71, 0.71, and 0.76, respectively **(** **Figure 2C** **)**. Nomogram calibration showed a good calibration performance in the training set as well as in the validation set **(** **Figures 2D, E** **)**. The Hosmer–Lemeshow test exhibited no statistical significance either in the training or validation sets, indicating a good agreement between the predictions based on nomogram and actual results. Decision curve analysis showed that the nomogram had better performance than clinical and radiomics models **(** **Figure 2F** **)**. DeLong’s test showed that the AUC between nomogram and clinical models exhibited statistical significance in the training set (*P* = 0.018); however, no statistical significance was found in the validation set (*P* = 0.433). The nomogram model did exhibit the best predictive performance among the three models, both in the training and validation sets.

#### Comparison of Progression-Free Survival of Second-Line Treatment Between Patients With or Without Acquired T790M Mutation

**Figure 3:**
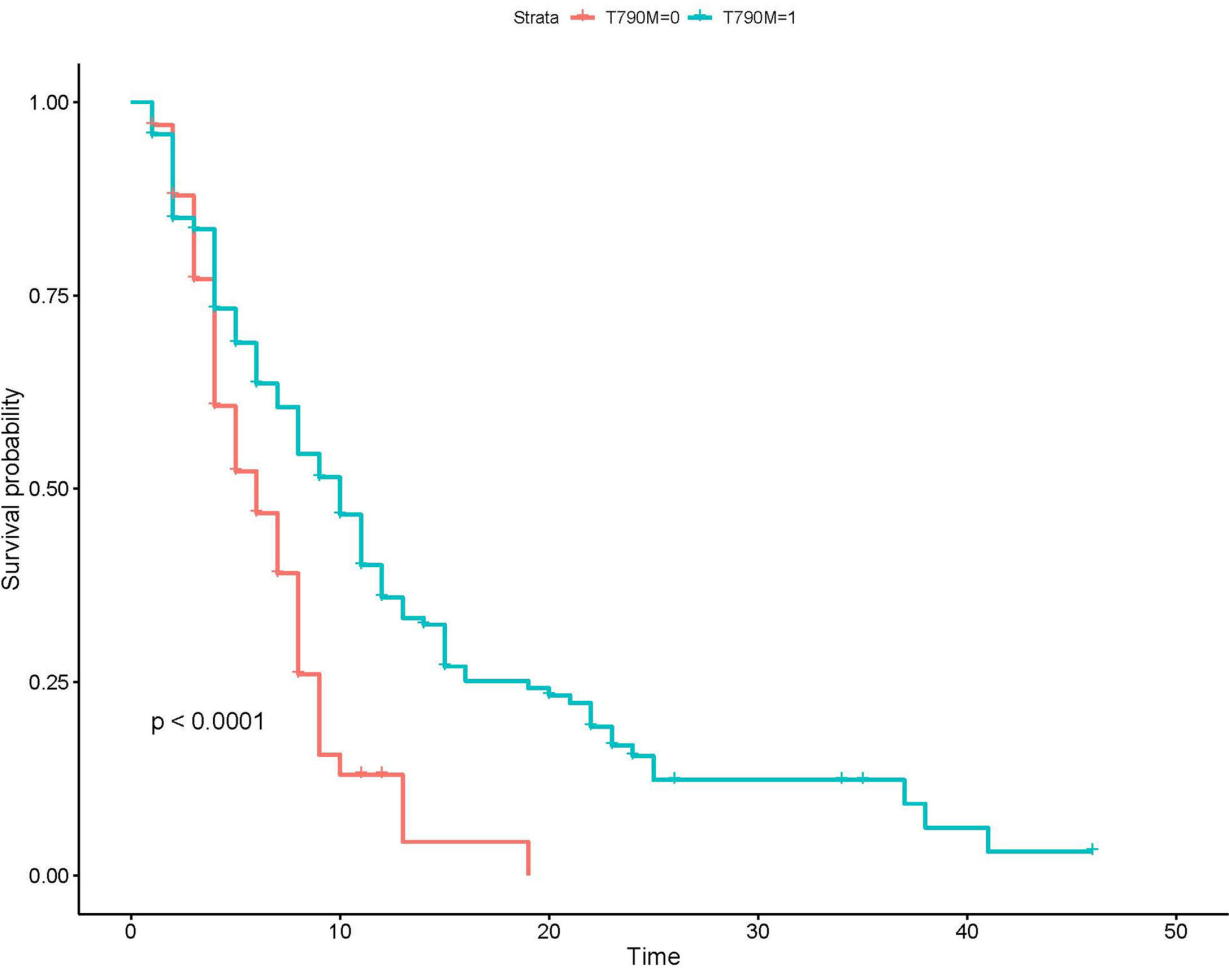
Kaplan–Meier analysis of patients with different T790M mutation status. T790M-0, the group without acquired T790M mutation; T790M-1, the group with acquired T790M mutation.

Of all the143 patients with acquired T790M mutation treated with osimertinib, 111 patients developed disease progression at the last follow-up; the median PFS was 9 months (95%CI: 7.0–10.0). Of the 67 patients without acquired T790M mutation treated with platinum-based chemotherapy, 61 patients developed disease progression at the last follow-up; the median PFS was 5 months (95%CI: 4.0–6.0). Kaplan–Meier analysis showed that the PFS of patients with acquired T790M mutation who received osimertinb were significantly longer than the PFS of patients without T790M mutation who received chemotherapy **(** **Figure 3** **).**

### Performance of Radiomics in the Prediction of Progression-Free Survival of Osimertinib Treatment

#### Prognostic Value of Clinical Factors

Among 143 patients treated with osimertinib, univariate Cox regression showed that 3 clinical factors including age, performance score and first-line TKIs were associated with prognosis. Multivariate analysis showed that age and first-line TKIs were independent clinical indicators of PFS. The hazard ratios for age and first-line TKIs were 0.98 (95% CI: 0.95–1.00, P= 0.034), 1.36 (95% CI: 1.03-1.78, P = 0.028), respectively. The predictive ability of the clinical model was limited. Kaplan–Meier analysis is displayed at **Supplementary Material 2**.

#### Prognostic Value of Radiomic Features

After univariate Cox regression and LASSO Cox regression, 12 features were retained and finally introduced into the radiomics model. LASSO cox regression was displayed at **Supplementary Material 3**. By using Kaplan–Meier curve analysis, radscore, which stratified patients into the low- and high-risk groups, exhibited significant PFS differences in the training set (*P* < 0.0001); however, no statistical significance was observed in the validation set (*P* = 0.091). Kaplan–Meier analysis was displayed at **Supplementary Material 4**. The formula for calculating radscore was presented as follows:


$$\begin{array}{c} \text{Radscore }=\;0.139*\text{ShortRunLowGreyLevelEmphasis}\_\text{AllDirection}\_\text{offset}4\_\text{SD}+\\ 0.070*\text{LongRunLowGreyLevelEmphasis}\_\text{AllDirection}\_\text{offset}4\_\text{SD}+\\ 0.042*\text{InverseDifferenceMoment}\_\text{AllDirection}\_\text{offset}1\_\text{SD}+\\ 0.037*\text{GreyLevelNonuniformity}\_\text{AllDirection}\_\text{offset}4\_\text{SD}+\\ 0.025*\text{GLCMEntropy}\_\text{AllDirection}\_\text{offset}1\_\text{SD}+-\\ 0.001*\text{ClusterProminence}\_\text{angle}135\_\text{offset}4+-\\ 0.017*\text{GLCMEntropy}\_\text{angle}45\_\text{offset}7+-\\ 0.067*\text{HighGreyLevelRunEmphasis}\_\text{angle}0\_\text{offset}1+-0.080*\text{ShortRunEmphasis}\_\text{angle}135\_\text{offset}1+-\\ 0.095*\text{ShortRunEmphasis}\_\text{angle}0\_\text{offset}1+-\\ 0.129*\text{SmallAreaEmphasis}+-\\ 0.136*\text{ClusterProminence}\_\text{AllDirection}\_\text{offset}1\_\text{SD} \end{array}$$

#### Prognostic Value of Nomogram Model

**Figure 4:**
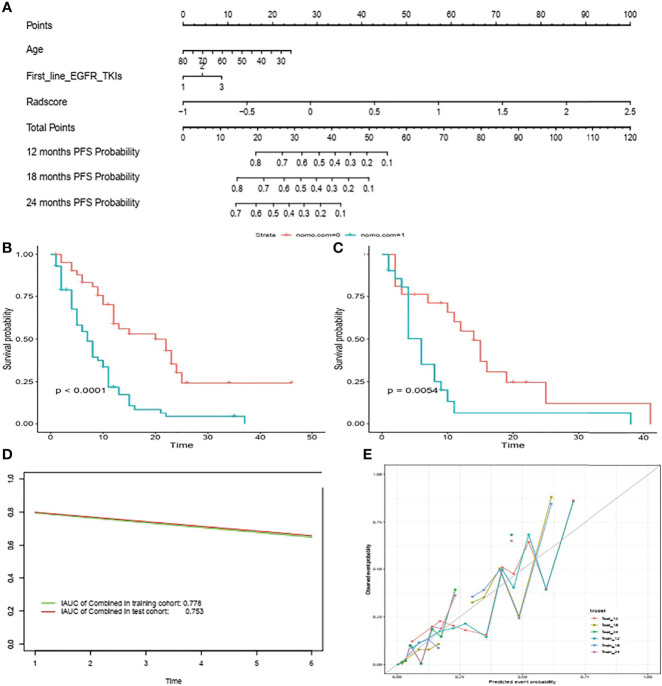
Nomogram for predicting PFS in patients with acquired T790M mutation treated with osimertinib **(A)** Risk stratification in the training set **(B)** and validation set **(C)**. Time-dependent ROCs were plotted in the training as well as validation sets at 6 months **(D)** Calibration curves of the nomogram to validate the 12m, 18m and 24m PFS rate in the training set and validation set **(E)**.

The selected clinical factors including age and first-line EGFR TKIs and the above-mentioned 12 radiomic features were integrated to build a nomogram model **(** **Figure 4A** **)**. The C-index of the combined nomogram model achieved the best prediction for PFS among the three models both in the training set and validation set, as displayed in **Table 2**. The combined nomogram could achieve better risk stratification both in the training set and the validation set (**Figures 4B, C**). Time-dependent ROCs were plotted in the training and validation sets at 6 months **(** **Figure 4D** **),** whereas calibration curves demonstrated a poor agreement between the predicted probability and the observed probability both in the training set and the validation set **(** **Figure 4E** **).**

**Table 2:** The performance of three models for progression-free survival prediction in patients treated with osimertinib.

Model	C-index (95%CI)	
	Training cohort	Validation cohort
Clinical	0.612 (95%CI: 0.530–0.694)	0.597 (95%CI: 0.474–0.720)
Radiomics	0.605 (95%CI: 0.534–0.680)	0.610 (95%CI:0.508–0.712)
Combined	0.686 (95%CI: 0.621–0.751)	0.630 (95%CI:0.514–0.746)

## Discussion

In this study, we combined clinical factors and radiomic features based on contrast-enhanced CT images to predict acquired T790M mutation and survival in advanced lung adenocarcinoma patients with progression after first-line EGFR TKI–targeted therapy. Our observations revealed that radiomic features may help to identify the status of acquired T790M mutation but might have a limited value in predicting the PFS of lung cancer treated with osimertinib after first-line EGFR TKI failure. When radiomic features were combined with clinical factors, the nomogram model outperformed clinical and radiomics models both in predicting the T790M mutation status and survival.

We found that patients with exon 19 deletion were more likely to develop acquired T790M mutation than those with exon 21 L858R mutation or other rare type of mutation, which was in accordance with recent studies (15, 16). We also found that acquired T790M mutation was associated with the PFS of first-line TKIs. The longer exposure to EGFR TKIs, the larger the possibility to develop acquired T790M mutation. This finding was in line with Kawamura T’ s research (17). The finding that patients exhibiting initial exon 19 deletion and longer PFS of first- or second-generation TKIs were more likely to benefit from osimertinib treatment can be useful for clinicians’ treatment decision-making. Furthermore, the result of our study showed that patients with a lower N stage have a larger likelihood to develop acquired T790M mutation, suggesting that regional lymph node progression may not be the reason for developing acquired T790M mutation, whereas Ouyang W et al. found that distant lymph node metastasis is an independent risk factor of acquired T790M mutation (18).

The research of Rossi G et al. showed the potential of radiomic features extracted from pre-treatment CT in predicting acquired T790M mutation; however, the small sample of the study hindered further investigation. In our study, CT images after progression on TKIs were chosen for analysis based on two considerations: 1) the resistance mechanism that might be reflected in radiomic features has already occurred, and 2) patients have stopped taking first- or second-generation TKIs once progression is confirmed, and TKIs would exert no more influence on radiomic features. Our results indicated that radiomics alone could achieve a similar efficiency as clinical factors did in predicting acquired T790M mutation in advanced lung adenocarcinoma patients with progression on first- or second-generation EGFR TKIs. When CT radiomics is combined with clinical factors, the prediction efficiency may further improve. In clinical practice, a rebiopsy is still a gold standard to identify the resistance mechanism of first-line TKI treatment failure. However, the nomogram displayed the possibility of being served as a complementary tool to predict acquired T790M mutation. For patients who are unable to undertake a rebiopsy or with the negative T790M results of liquid biopsy, a non-invasive nomogram can play an important role in selecting candidates for osimertinib treatment.

Our study suggested that patients with acquired T790M mutation who received osimertinib have longer PFS than those without acquired T790M mutation who underwent chemotherapy. The result can possibly be explained by the indolent nature of the growth of the T790M cells (19).

Patients’ age and first-line TKIs were related to the PFS of second-line osimertinib treatment in this study. However, Tang X et al. found that the performance score and M stage were independent indicators for survival in metastatic NSLCL patients with T790M mutation treated with osimertinib (12). Furthermore, Chen Y et al. showed that the coexisting 19 del and T790M, M stage and biopsy specimen are correlated with the survival of patients treated with osimertinib (20). Our result showed inconsistency with the above-mentioned studies. The reasons of first-line TKIs associated with the PFS of osimertinib treatment may lie in two aspects: 1) the incidence of acquired T790M mutation among icotinib, gefitinib, or erlotinib ranged from 53.3% to 72.9% in our study, suggesting that patients who had gefitinib or erlotinib exposure are inclined to develop T790M mutation and therefore benefit from osimertinib; 2) the abundance of T790M mutation may vary from the above-mentioned three TKIs, leading to a different treatment response. In our study, a younger age is related to a favorable outcome, as with Ono T’s results (21). However, the inconsistency of the relationship between age and the prognostic value existed among various researches in which age is unrelated to prognostic survival or a younger age is an unfavorable clinical factor of prognosis (12, 22).

Treatment response differs among patients with advanced lung adenocarcinoma harboring acquired T790M mutation. Thus, identifying patients who will have a longer PFS of osimertinib is crucial to maximize the effectiveness at a relatively low cost. Several studies explored the efficiency of CT radiomics in predicting survival in advanced lung cancer patients who received EGFR-TKIs and showed promising results (10, 12). In our study, clinical, radiomics, and combined nomogram models were constructed to predict the prognosis of patients treated with second-line osimertinib. The clinical and radiomics models achieved a similar prognostic value in both the training set and the validation set, while combined nomogram achieved the highest prognostic value among the three models.

There were some limitations of this study. First, the retrospective nature makes it unable to avoid selection bias. A lack of external validation also left the robustness of the nomogram to be verified, further studies should be focused on big data, multicenter study. However, our results did show the potential of CT radiomics in the prediction of acquired T790M mutation and the PFS on third-generation TKIs. Last but not the least, the number of enrolled patients was relatively small.

In conclusion, our findings revealed the possibility of identifying acquired T790M mutation by CT radiomic features after progression on first- or second-generation of TKIs. Also, our study showed that CT radiomics might have a limited value in identifying the rapid and slow progression of patients harboring acquired T790M mutation treated with osimertinib.

## Data Availability Statement

The original contributions presented in the study are included in the article/**Supplementary Material**. Further inquiries can be directed to the corresponding authors.

## Ethics Statement

The studies involving human participants were reviewed and approved by The Medial Ethnical Committee of Hunan Cancer Hospital. Written informed consent for participation was not required for this study in accordance with the national legislation and the institutional requirements.

## Author Contributions

All of the coauthors listed meet the criteria for authorship. XPY and XHY were involved with study concept and design. CF was responsible for collecting data. CL and MG contributed to data processing. CF, CL, and XHY were involved with drafting/revising the manuscript together. XCY and HL were involved with analysis and interpretation of data. XPY was responsible for manuscript revision. XPY and KL were responsible for study supervision. All authors contributed to the article and approved the submitted version.

## Funding

This work was supported by the National Natural Science Foundation of China (82003867), Scientific Research project of Hunan Health Commission (202213015439) and Natural Science Foundation of Hunan Province (2022JJ40245).

## Conflict of Interest

Author HL was employed by General Electric (GE) Healthcare.

The remaining authors declare that the research was conducted in the absence of any commercial or financial relationships that could be construed as a potential conflict of interest.

## Publisher’s Note

All claims expressed in this article are solely those of the authors and do not necessarily represent those of their affiliated organizations, or those of the publisher, the editors and the reviewers. Any product that may be evaluated in this article, or claim that may be made by its manufacturer, is not guaranteed or endorsed by the publisher.
