# Supplementary material for: Can CT Radiomics Detect Acquired T790M Mutation and Predict Prognosis in Advanced Lung Adenocarcinoma With Progression After First- or Second-Generation EGFR TKIs?
Source: Front Oncol. 2022 Jul 6;12:904983. doi: 10.3389/fonc.2022.904983 (PMC9300753; doi:10.3389/fonc.2022.904983)

Supplementary Material

# Supplementary Figures

**Figure 1** LASSO regression assessing the predictive ability of radiomic features in identifying acquired T790M mutation.


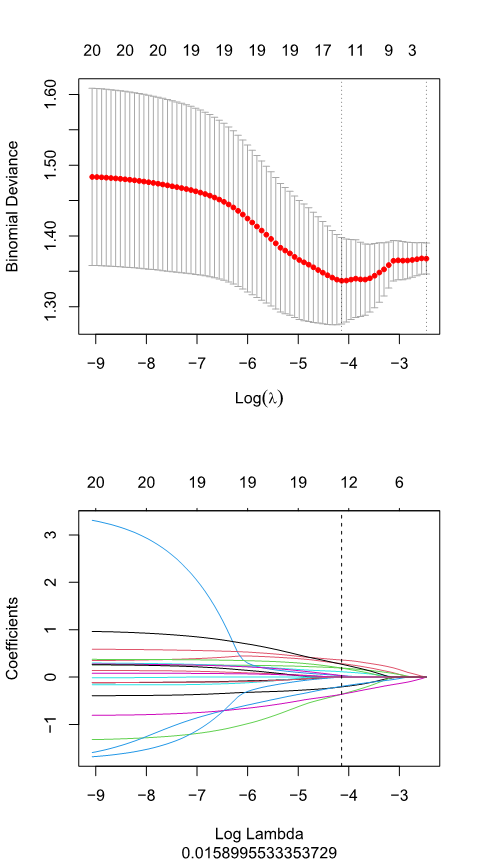


**Figure 2** The ablility of risk stratification of clinical model in the training set and validation set.


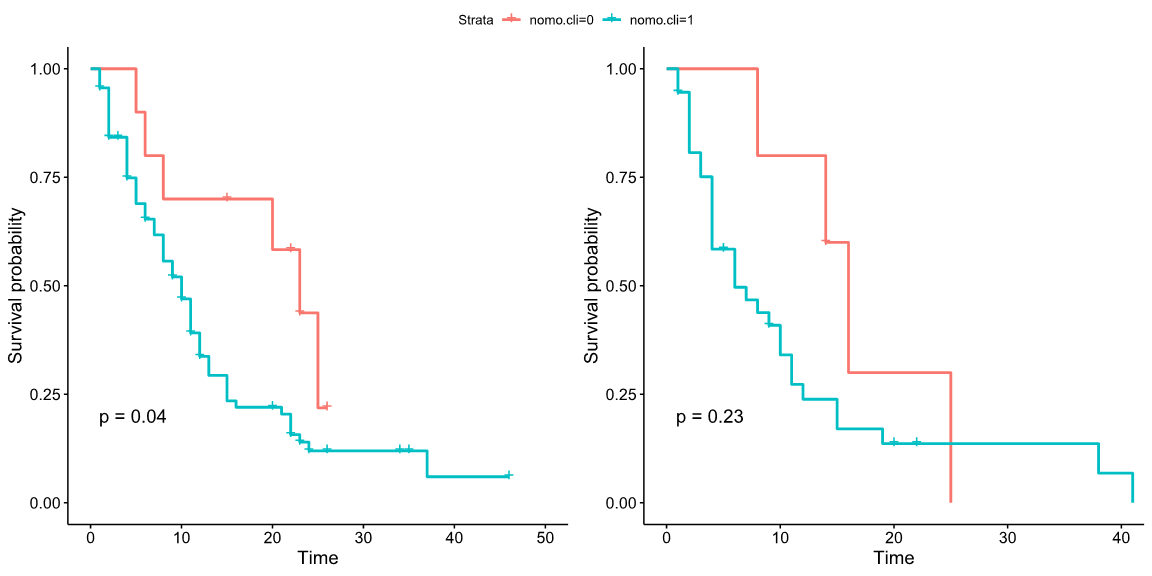


**Figure 3** LASSO cox regression assessing the prognostic value of radiomic features in patients with acquired T790M mutation received osimertinib therapy.

**
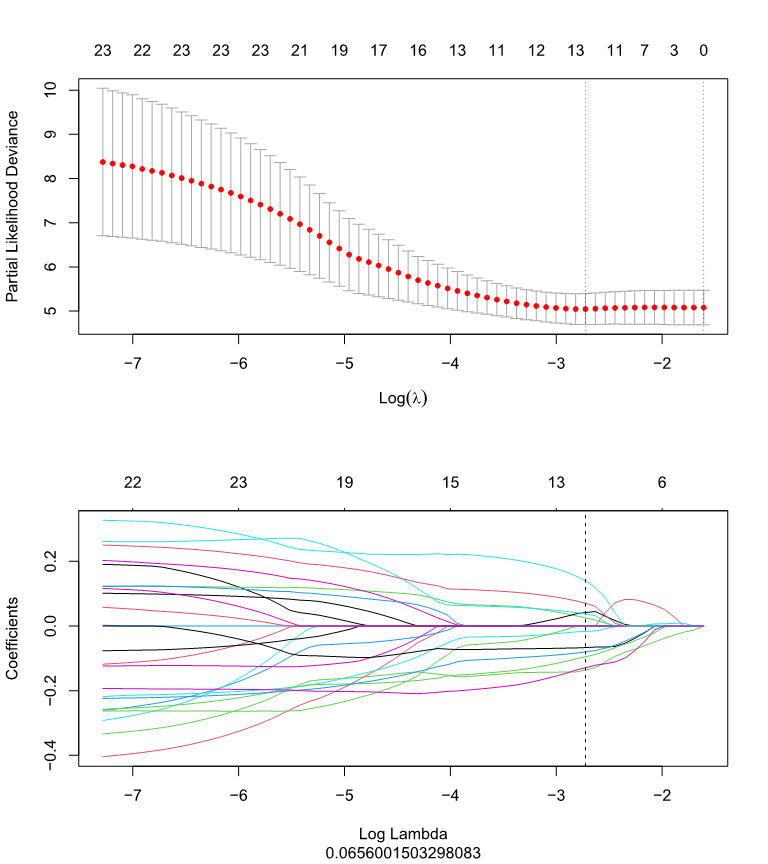
**

**Figure 4** The ability of risk stratification of radiomics model in the training set and validation set.


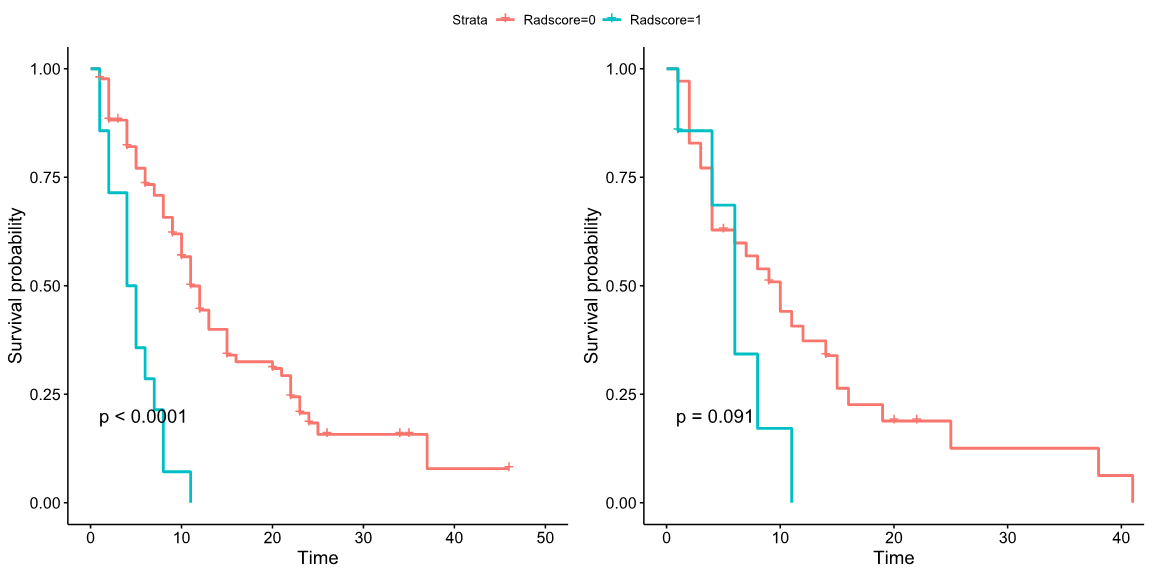

Supplement: Supplementary file 1 [file DataSheet_1.docx]
